# Synthesis, Spectroscopy and Electrochemistry of Fe(II) and Fe(III) Quinonemonooxime Complexes and Their DNA Cleaving Activities

**DOI:** 10.1155/MBD.1999.81

**Published:** 1999

**Authors:** Anupa Murugkar, Subhash Padhye, Deepti Deobagkar

**Affiliations:** 1 Department of Chemistry University of Pune Pune 411 007 India; 2 Department of Zoology University of Pune pune 411 007 India

## Abstract

Iron(II) and iron(III) complexes of 3,5-di-tert-butyl-o-benzoquinonemonooxime were synthesized and characterized by spectroscopic and electrochemical studies. Their ability to cleave DNA has been investigated under aerobic conditions at room temperature and in the presence and absence of H_2_ O_2_. The plasmid DNA pBR322 was effectively cleaved by these complexes in a concentration dependant manner.


### SYNTHESIS, SPECTROSCOPY AND ELECTROCHEMISTRY OF Fe(II) AND Fe(III) QUINONEMONOOXIME COMPLEXES AND THEIR DNA CLEAVING ACTIVITIES

Anupa Murugkar[1], Subhash Padhye*[1] and Deepti Deobagkar[2]

[1] Department of Chemistry, University of Pune, Pune-411 007, India
[2] Department of Zoology, University of Pune, Pune-411 007, India

**Abstract:**

Iron(II) and iron(III) complexes of 3,5-di-tert-butyl-o-benzoquinonemonooxime were synthesized and characterized by spectroscopic and electrochemical studies. Their ability to cleave DNA has been investigated under aerobic conditions at room temperature and in the presence and absence of $H_2O_2$. The plasmid DNA pBR322 was effectively cleaved by these complexes in a concentration dependant manner.

**Introduction:**

The reagents which are capable of controlled DNA and RNA-sequence specific cleavages have been employed as tools in molecular biology. For example, they are used as footprinting agents,[1] as probes for detecting structural variations in DNA and RNA[2], as artificial nucleases[3] or as compounds capable of targeting aberrant DNA base sequences for occlusion by direct binding at DNA or mRNA.[4] The sequence specific cleavages of DNA are, however, limited in terms of the specificities and availabilities of natural restriction endonucleases. One approach to overcome this difficulty has been to append DNA-recognizing molecules with a reagent capable of chemical cleavages of DNA. Fe(II)-EDTA complex has commonly been used for such a purpose by attaching it to oligonucleotides[5,6], intercalators[7] or a combination thereof.[8] Numerous examples of metal complexes requiring oxidising agents to induce strand scission are available in literature.[9,10] The major classes include Cu(I)-bis-(1,10-phenanthroline)[1c,11-14] compounds, metal-porphyrin complexes[15-22] and rhodium complexes.[23] Additionally, the octahedral complexes of ruthenium and cobalt with 1,10-phenanthroline[24] and bipyridyl ligands[25] constitute the photochemical DNA cleaving agents so does the uranyl acetate.[26]

Specific examples include the Mn(II)-bleomycin compound which can degrade DNA in the presence of $H_2O_2$[27], although, the precise mechanism of such cleavage is not known. Nishida et al[28] have observed that a dinuclear Mn(IV) species $[Mn_2O_3L_2]^{2+}$ , where L=1,4,7-trimethyl-1,4,7-triazacyclononane also exhibits a high activity for nicking the plasmid DNA (pBR322) in the presence of $H_2O_2$ where the active species is postulated to be a peroxide adduct of the Mn(IV) complex. The ability of Cu(II)-GSH (where GSH is the reduced form of glutathione) to induce single strand breaks in supercoiled DNA at very low concentrations has been described by Reed and Douglas.[29] The diiron complex $[Fe_2$(N,N,N'N'-tetrakis-(2-benzimidazolylmethyl)-2-hydroxy-1,3diaminopropane) (OH) $(NO_3)_4]$ is found to induce cleavages of double strands of pBR322 in the presence of $O_2$ or $H_2O_2$ probably through a hydrolytic mechanism while a group of redox active co-ordination compounds have been shown to cleave the same by phosphodiester bond hydrolysis.[30] The sequence specific DNA cleavage especially at the AT rich regions by $Cu(TAAB)^{2+}$, where TAAB is tetrabenzo [1,3,9,13]tetraaza cyclohexadecine, has recently been reported by Durackova et al.[31]

Quinones constitute a vast family of redox-active compounds both natural as well as synthetic which can act as co-ordinating ligands for a large .number of metal ions. They are present ubiquitously in plants, bacteria and animals and have wide ranging functions from participation in electron transfer reactions to defending organisms against insect attack or bacterial infections.[32] Some of the quinone derivatives which include adriamycin, mitomycin, streptonigrin and lapachols are also used as antiproliferative compounds which exert their action through inhibition of DNA synthesis. However, the intracellular targets of these compounds is still a matter of investigation.[33].Some of the biologically active quinone ligands undergo facile metal complexation reactions[34] and these have been found to influence the biological activities considerably. In the present work we report on the results obtained on the DNA cleavage activities of iron(II) and (III) compounds of 3,5-di-tert-butyl-o-benzoquinonemonooxime on a plasmid DNA pBR322 in the presence of an oxidant.

**Materials and Methods**

Analytically pure grade chemicals were obtained as indicated $FeCl_2$, $FeCl_3$ (anhydrous) and TRIS base were from Fluka; EDTA and SOD from Sigma Chemical Company; Sodium Azide from Riedel-de-haen, Hanover (Germany); glycerol form CDH and HPLC grade DMSO from Spectralab were used. The plasmid DNA pBR322 was purchased from Bangalore Genei (India). Electrophoretic agarose was the product of Sisco Research Laboratories, Bombay, India.

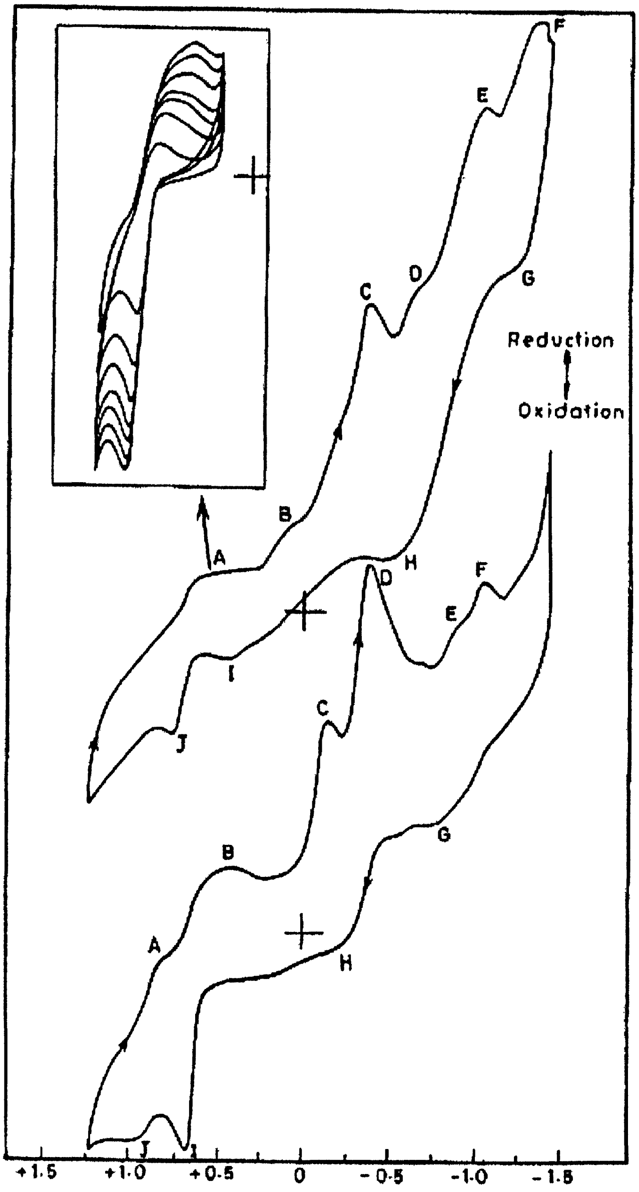

Figure 1. Cyclic voltammograms (lOOmV/S) of $10^{-3}$ M solutions of (a) $Fe^{II}(L)_2Cl$ and (b)$Fe^{III}(L)_3Cl$ in DMSO (0.1 M $Et_4NC1O_4$) with inset showing the scan rate dependence (mV/S) for the peak centered at +0.70 V.

IR spectra of the complexes were recorded on a Perkin-Elmer 400 IR spectrophotometer while the UV-VIS spectra were recorded on a Shimadzu 90 UV-spectrophotometer. Cyclic voltammograms were obtained on the Bioanalytical Laboratory System BAS CV-27 in DMSO using 0.1 M TEAP as the supporting electrolyte and a three electrode system comprising of a Pt working electrode, Saturated Calomel Electrode as the reference electrode and Pt wire as an auxillary electrode.

**Experimental**

**Synthesis of Ligand and Metal Complexes.**

Synthesis and purification of the ligand, 3,5-di-tert-butyl-o-benzoquinonemonooxime, was carried out following a general method for the synthesis of oximes.[35] Its iron (II) and (III) compounds were synthesized by reacting the methanolic solutions of ligand with the aqueous solutions of metal ions in appropriate stoichiometric ratio under reflux for 3 hrs. The precipitates obtained after setting aside the reaction mixture overnight were filtered, washed with cold water and dried in vacuum.

### Results and Discussion

The iron complexes were assigned the formulations as $Fe^{II}(L)_2Cl$ and $Fe^{III}(L)_3Cl$ for compounds **1** and **2** respectively based on their elemental analyses. The assignments of their IR bands are listed in **Table 1**. The free ligand shows a broad band at 3221 $cm^{-1}$ which can be assigned to the intramolecular hydrogen bonding involving the oximino hydroxyl group. This absorption is lost on metal complexation indicating the replacement of H by the corresponding metal ions. The N-O stretching vibration at 1528 $cm^{-1}$ in the free ligand is shifted to 1493 and 1482 $cm^{-1}$ respectively on complexation while the.ν(C=N) and ν(C=O) frequencies are shifted towards lower frequency during complexation and appear at 1600 and 1560 $cm^{-1}$ respectively. The out-of-plane OH deformations appear at 888, 849 and 847 $cm^{-1}$ respectively in the free ligand and its iron complexes.

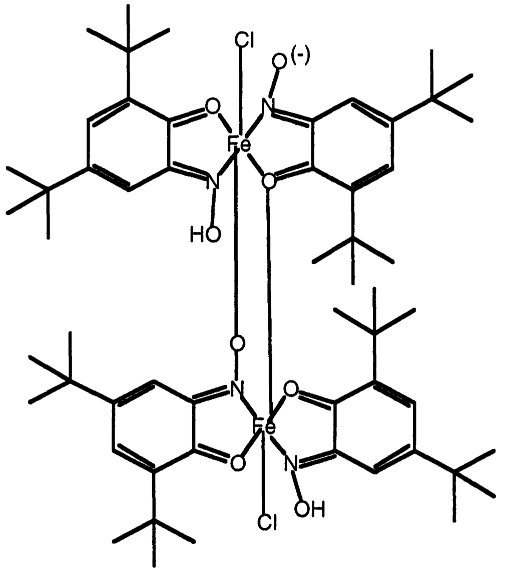

$[Fe(L_2)Cl]$ (**1**)

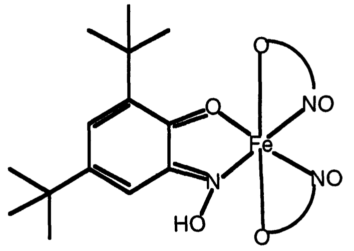

$[Fe(L_3)]Cl$ (**2**)

**Table 1.** Elemental analysis, spectroscopic, magnetic and cyclic voltammetric data for Compounds **1** and **2**

| Compound | Elemental Analysis %C | %H | %M | $\mu_{(298)}$ B.M. | Significant IR bands[a] (C=O) | (C=N) | (N-O) $cm^{-1}$ | UV-VIS[b] $cm^{-1}$ | $E_{1/2}$[b](V) $M^{2+}/M^{3+}$ |
|---|---|---|---|---|---|---|---|---|---|
| 1. $Fe^{II}(L)_2Cl$ | 59.95 (61.48) | 7.40 (7.57) | 14.23 (13.84) | 2.3 | 1603 | 1559 | 1493 | 22471,19305 15267,14084 | +0.70 |
| →2. $Fe^{III}(L)_3Cl$ | 64.87 (65.02) | 7.78 (7.76) | 8.81 (7.65) | 4.2 | 1600 | 1560 | 1482 | 22471, 14900 | +0.58 |

→ [a]As nujol mulls; [b]In DMSO; Values in parentheses represent calculated values.

The electronic spectra of the complexes exhibit ligand to metal charge transfer bands at 22471 $cm^{-1}$ while their d-d transitions are observed in the range 14000 to 19500 $cm^{-1}$ region. The lowered magnetic moments of the complexes have been explained on the basis of antiferromagnetic interactions between the central metal atom and the bound semiquinone radical anions as found in the case of iron (III) complexes of phenanthrenequinone and tetrachlorobenzoquinone ligands.[36] The cyclic voltammograms of both the iron complexes are shown in **Figure 1** which indicate several reduction (A,B,C,D,E & F) and oxidation peaks (G,H,I & J). Repeated scans of the complexes at different scan rates show that the main reduction peak A and its counterpart J (complex **a**) and peaks B and I (complex **b**) are fully reversible corresponding to the $M^{2+}/M^{3+}$ metal couple as shown in **Table 1**. The shift to higher positive potential seems to result in the decreased DNA cleavages. Peaks A and J in complex b correspond to the reduction-oxidation peaks of the halide (chloride) ligand.[37] Rest of the peaks correspond to ligand with slight shift in their peak potentials after complexation. An irreversible peak at -1.05 V Vs SCE can be assigned to the reduction of the imine function.[38]

In the present work circular double stranded plasmid DNA, viz. pBR322, was used to investigate the DNA cleavages by the iron complexes under aerobic conditions. The reaction products were analyzed by conventional electrophoretic technique and the patterns of DNA cleavages are shown in **Figure 2**. The results clearly show that in the presence of $H_2O_2$ at 10 and 20 μM of metal-complex concentrations there is a stepwise conversion of form I (supercoiled) to form II (nicked or open circular) predominantly. Such cleavages have been known to proceed through the formation of OH· and $O_2^{-\cdot}$ radicals. A careful examination of the cleavage pattern in **Figure 2** reveals an additional band with considerable intensity just below the nicked circular band which may be arising out of the conformational changes in DNA leading to its altered electrophoretic mobility on the gel. Further experiment on the linearized pBR322 with and without $H_2O_2$ showed the absence of any additional band although the electrophoretic mobility of the bands treated with $H_2O_2$ was changed. It suggests that the binding of present iron complexes might be inducing nicking of supercoiled DNA in addition to some conformational changes rather than acting specifically on the linear form of DNA.

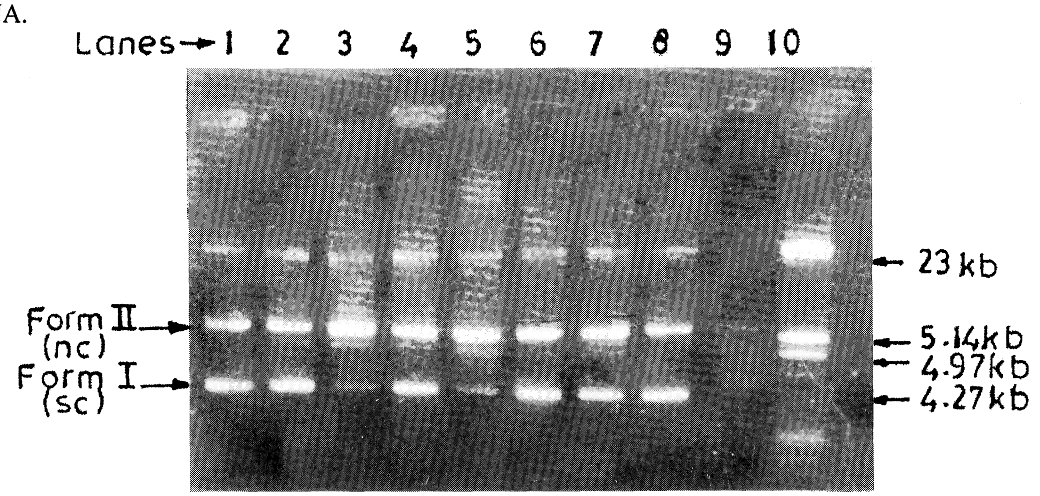

**Figure 2.** 1% Agarose gel showing the results of electrophoresis of pBR322 plasmid DNA (~4.3 kb); DNA (300 ng); metal complexes (10μM and 20μM) in DMF; $H_2O_2$ (2 μl) incubated at 37°C for 30 min. Autoclaved distilled water was used to make final volume 20 μl.

Lane 1 DNA; Lane 2 DNA + **1** (10μM); Lane 3 DNA + **1** (10μM) + $H_2O_2$; Lane 4 DNA + **1** (20 μM); Lane 5 DNA + **1** (20 μM) + $H_2O_2$; Lane 6 DNA + **2** (10 μM); Lane 7 DNA + **2** (10 μM) + $H_2O_2$;.Lane 8 DNA + **2** (20 μM); Lane 9 DNA + **2** (20 μM) + $H_2O_2$; Lane 10. λ marker.

In order to find out the type of radical species responsible for the DNA cleavages another experiment using various radical scavengers such as glycerol, DMSO (as OH· radical scavengers), →sodium azide (as $O_2$ scavenger) as well as SOD (as $O_2^{-\cdot}$ radical scavenger) with and without the oxidant under identical reaction conditions was undertaken. Since none of these scavengers were found to provide protection against the DNA degradation the most probable causative species for the DNA cleavages are thought to be the semiquinone radical anion species generated by the redox-active quinonoidal ligands as pointed out by Pierpont et al.[39] Such metal-semiquinone compounds inducing DNA nicks and cleavages have earlier been observed in case of the antiproliferative quinone compounds like adriamycin and mitomycin.[33]

**Acknowledgements**
AM would like to acknowledge financial assistance from CSIR, New Delhi (India).

**References**
1. (a) Tullius T.D., *Nature*, 1988,**332**,663.
(b) Tullius T.D., Dombroski B. A., *Science*, 1985,**230**,679.
(c) Hertzberg R. P., Dervan P.B., *Biochemistry*, 1984,**23**,3934.
(d) D'Andrea A. D., Haseltine W.A., *Proc. Natl. Acad. Sci. U. S. A.*, 1978,**75**,3608.
2. (a) Kuwabara M. B., Sigman D.S., *Biochemistry*, 1987, **26**, 7234.
(b) Mazumder A., Chen C-H B., Gaynor R.B., Sigman D.S., *Biochem. Biophys. Res. Commun.*, 1992, **187**, 1503.
(c) Pearson L., Chen C-H B., Gaynor R..P., Sigman D.S., *Nucleic Acids Res.*, 1994, **22**, 2255.
3. (a) Sigman D.S., *Acc.Chem.Res.*,1986,**19**,180.

(b) Baaile L.A., Barton J.K., *J. Am. Chem. Soc.*, 1987,**109**,7548.
4. Francis K.C., Cummins D., Oakes J., *J. Chem. Soc. Dalton Trans.*, 1985,493.
5. Boutorin A.S., Vlassov V.V., Kazakov S.A., Katiann I. V., Podyminogin M. A., *FEBS Lett.*, 1984,**172**,43.
6. Dreyer G. B., Dervan P.B., *Proc. Natl. Acad. Sci. USA*, 1985,**82**,968.
7. Hertzberg R. P., Dervan P.B., *J.Am.Chem.Soc.*,1982,**104**,313.
8. Blodet-Forget M., Chassignol M., Takasugi M., Thuong N. T., Helene C., *Gene*, 1988,**72**, 361.
9. (a) Barton J.K., Raphael A.L.,*J.Am.Chem.Soc.*,1984,**106**,2466.
(b) Sagripanti J.L., Kraemer K.H.,*J.Biol.Chem.*,1989,**264**,1729.
10. Yamamoto K., Inoue S., Yamazaki A., Yoshinaga T., Kawanishi S., *Chem. Res. Toxicol.*, 1989,**2**,234.
11. (a) Downey K.M., Que B. G., So A.G., *Biochem.Biophys.Res.Commun.*,1980,**92**,264.
(b) Chu B.F., Orgel L.E., *Proc.Natl.Acad.Sci.USA*, 1985,**82**,963.
12. Francois J.C., Saison-Behmoaras T., Chassignol M., Thuong N. T., Helene C., *J.Biol. Chem.*,1989,**264**,5891.
13. (a) Spassky A., Sigman D.S., *Biochemistry*,1985,**24**,8050.
(b) Thederahn T.B., Kuwabara M.D., Larsen T.A., Sigman D.S., *J.Am.Chem.Soc.*,1989, **111**,4941.
14. Veal J.M., Rill R.L.,*Biochemistry*,1989,**28**,3243.
15. Lown J.W., Joshua A.V.,*J.Chem.Soc.Chem.Commun.*,1982,1298.
16. Lown J.W. Sondhi S.M., Ong C.W., Skorobogaty A., Kishikawa H., Dabriowak J.C.,*.Biochemistry*,1986,**25**,5111.
17. Bernadou J., Lauretta B., Pratviel G., Maunier B., *C.R.Acad.Sci.Ser.3*,1989,**309**,409.
18. Groves J.T., Farrell T.P.,*J.Am.Chem.Soc.*,1989,**111**,4998.
19. Ward B.,Skorobogaty A., Dabriowak J.C., *Biochemistry*,1986,**25**,6875.
20. Le Doan T., Perrouault L., Helene C., Chassignol M., Thuong N.T.,*Biochemistry*, 1986,.**25**,6736.
21. Arounaguiri S.,Maiya B.G., *Inorg.Chem.*, 1996,**35**,4267.
22. Mehta G.,Sambaiah T.,Maiya B.G., Shirish M., Dattagupta A., *J.Chem.Soc. Perkin Trans.I*,1995,295.
23. Kirshenbaum M., Tribolet R., Barton J.K.,*Nucleic Acids Res.*,1988,**16**,7943.
24. (a) Barton J.K.,*Science*,1986,**233**,727.
(b) Zelenco O., Gallagher J., Xu Y., Sigman D.S., *Inorg.Chem.*, 1998, **37**,2198.
25. (a) Neyhart G.A., Grover N. Smith S.R., Kalsbeck W.A., Fairley T.A., Cory M., Thorp H.H.,*J.Am.Chem.Soc.*,1993,**115**,4423.
(b) Neyhart G.A., Cheng Chien-Chung, Thorp H.H.,*J.Am.Chem.Soc.*,1995,**117**,1463.
(c) Goll J.G., Thorp H.H.,*Inorg.Chim.Acta*,1996,**242**,219.
(d) Welch T.W., Ciftan S.A., White P.S., Thorp H.H.,*Inorg.Chem.*,1997,**36**,4812.
26. Nielsen P.E., Jeppesen C., Buchardt O.,*FEBS Lett.*,1988,**235**,122.
27. Sigman D.S., Graham D.R., D'Aurora V., Stern A.M.,*J.Biol.Chem.*,1979,**254**,12269.
28. Kobayashi T., Tsuchiya K., Nishida Y.,*J.Chem.Soc.Dalton Trans.*,1996,2391.
29. (a) Reed C.J., Douglas K.T., *Biochemistry J.*,1991,**275**,601.
(b) Reed C.J., Douglas K.T.,*Biochem.Biophys.Res.Commun.*,1989,**162**,1111.
30. Schnaith L.M.T., Hanson R.S., Que L.Jr.,*Proc.Natl.Acad.Sci.USA*,1994,**91**,569.
31. (a) Durackova Z., Labudova O., Andrezalova L., Labuda J., Kollarova M.,·Weser U., *Int.J.Biochem.Cell Biol.*,1995,**27**,1341.
(b) Labudova O., Labuda J., Fenikova L., Kollarova M., Durackova Z.,*J.Inorg.Biochem.*,.1996,**61**,227.
32. Thomson R.H., In *Naturally Occurring Quinones*, 2nd Ed.,1971, Academic Press, London.
33. (a) Bachur N. R., Gordon S. L., Gee M. V., *Mol. Pharmacol.*, 1977,**13**,901.
(b) Crooke S.T., Reich S.D., In *Anthracyclines :Current Status and New Developments*, 1980, Academic Press, New York.
(c) Kenendy, K.A., Rockwell S., Sartorelli A. C., *Cancer Res.* 1980,**40**, 2356.
(d) Docampo R., Cruz F.S., Boveris A., Muniz R.P.A., Esquivel D.M.S., *Biochem. Pharmacol.*, 1970, *28*,723.
34. Kalyanraman E., Mason R.P., *Biochim.Biophys.Acta*, 1980, **630**, 119.
35. In *Vogel's Textbook of Practical Organic Chemistry*,(5th Ed.), Longman Science and Technology Inc., 1995,pp 1259.
36. (a) Buchanan R.M., Kessel S. L., Downs H.H., Pierpont C.G., Hendrickson D.N., *J. Am. Chem.Soc.*,1978,**100**,7894.
(b) Tuchagues J.P.M., Hendrickson D.N.,*Inorg.Chem.*,1983,**22**,2545.
(c) Que L. Jr., Kolanezyk R. C., White L.S.,*J.Am.Chem.Soc.*,1987,**109**,5373.
37. Kumbhar A.S., Padhye S.B., Saraf A.P., Mahajan H.B., Chopade B.A., West D.X., *Biol.Metals*, 1991,**4**,141.
38. Kumbhar A.S., Padhye S.B., West D.X., Liberta A.E.,*Trans.Met.Chem.*,1991,**16**,276.
39. Pierpont C.G., Lange C. W., *Prog. Inorg. Chem.*,(Ed. K. D. Karlin),John Wiley & Sons Inc., 1994,**41**,331 and references therein.

**Received: January 19, 1999 - Accepted: February 5, 1999 - Received in revised camera-ready format: March 4, 1999**

